# Mapping Single Walled Carbon Nanotubes in Photosynthetic Algae by Single-Cell Confocal Raman Microscopy

**DOI:** 10.3390/ma13225121

**Published:** 2020-11-13

**Authors:** Silvia Orlanducci, Gianluca Fulgenzi, Andrea Margonelli, Giuseppina Rea, Taras K. Antal, Maya D. Lambreva

**Affiliations:** 1 Department of Chemical Science and Technology, University of Rome ‘‘Tor Vergata’’, 00133 Rome, Italy; 2 Institute of Crystallography, National Research Council of Italy, 00015 Monterotondo Stazione, Italy; andrea.margonelli@ic.cnr.it (A.M.); giuseppina.rea@ic.cnr.it (G.R.); 3 Department of Molecular and Clinical Sciences, Faculty of Medicine and Surgery, Marche Polytechnic University, 60126 Ancona, Italy; gianluca.fulgenzi2@nih.gov; 4 Mouse Cancer Genetics Program, Center for Cancer Research, National Cancer Institute, Frederick, MD 21702-1201, USA; 5 Department of Biophysics, Faculty of Biology, Lomonosov Moscow State University, 119992 Moscow, Russian; taras_an@mail.ru; 6 Laboratory of Integrated Environmental Research, Pskov State University, 180000 Pskov, Russian

**Keywords:** Raman spectroscopy, single-walled carbon nanotubes, *Chlamydomonas reinhardtii*, fluorescence quenching, electron transfer, spectroscopic probes

## Abstract

Carbon nanotubes (CNTs) are among the most exploited carbon allotropes in the emerging technologies of molecular sensing and bioengineering. However, the advancement of algal nanobiotechnology and nanobionics is hindered by the lack of methods for the straightforward visualization of the CNTs inside the cell. Herein, we present a handy and label-free experimental strategy based on visible Raman microscopy to assess the internalization of single-walled carbon nanotubes (SWCNTs) using the model photosynthetic alga *Chlamydomonas reinhardtii* as a recipient. The relationship between the properties of SWCNTs and their biological behavior was demonstrated, along with the occurrence of excitation energy transfer from the excited chlorophyll molecules to the SWCNTs. The non-radiative deactivation of the chlorophyll excitation promoted by the SWCNTs enables the recording of Raman signals originating from cellular compounds located near the nanotubes, such as carotenoids, polyphosphates, and starch. Furthermore, the outcome of this study unveils the possibility to exploit SWCNTs as spectroscopic probes in photosynthetic and non-photosynthetic systems where the fluorescence background hinders the acquisition of Raman scattering signals.

## 1. Introduction

The latest advances in phyto-nanotechnology and plant nano-bionics brought to light the ability of single-walled carbon nanotubes (SWCNTs) to deliver into plants small molecules, fertilizers, or pesticides [1,2,3,4,5], to improve plant defense [6,7,8] or even to turn plant tissues into molecular sensors [9,10]. Experimental strategies using SWCNTs may also bare a capacity to improve productivity by increasing algal photosynthetic performance or their tolerance toward the unfavorable conditions taking place in mass production [11]. Unlike the increasing number of biomedical and agricultural applications of carbon nanotubes (CNTs), the use of nanomaterials in microalgal biotechnology is still in its nascent phase and very little is known about the mechanisms of cellular internalization of CNTs in unicellular photosynthetic microorganisms and on the relationship between the CNT physicochemical properties and their biological behavior in the cell. A key challenge, relevant to risk-assessment studies in algal toxicology, is the lack of straightforward and affordable methods for monitoring the cellular uptake of CNTs, which motivated the present study. So far, the detection of SWCNTs in plant cells rely mainly on fluorescence microscopy and exploits either labeling of nanotubes with fluorescent tags [12,13,14] or the intrinsic fluorescence of the SWCNTs in the near-infrared (NIR) region [6,15]. These methods enabled both tracing the spatiotemporal behavior of SWCNTs and their localization inside plant cells. However, their wide use remains limited by the need of sophisticated instrumentation, labeling of the CNTs with fluorescence dyes or laborious sample preparation.

Confocal Raman spectroscopy allows for direct and label-free imaging of CNTs inside the cells exploiting the strong Raman signal originating from the different vibrational modes of the nanotubes [16]. Raman scattering has been used for identification of CNTs in mammalian [17] and yeast cells [18], while only limited attempts are reported in an intact photosynthetic sample [19]. The applications of Raman spectroscopy and imaging to photosynthetic samples is generally limited by the strong fluorescence emission of the cellular fluorophores (mainly chlorophylls) that significantly outdoes the signal of vibrational Raman scattering spoiling its detection [20,21]. This problem may be bypassed by using Raman spectroscopy with a long-wavelength-excitation laser [22], which bares the risk of loss of sensitivity and thermal degradation of the biological specimen, or exploiting highly sophisticated and technologically advanced methods, such as non-linear coherent anti-Stokes Raman scattering (CARS), stimulated Raman scattering (SRS), and surface-enhanced Raman spectroscopy (SERS) [23]. A common experimental tactic used to drown the fluorescence of plant-type samples exploits strong pre-illumination of the analyzed spot, which saturates and vanishes (photo bleach) the fluorescence emission of the photosynthetic pigments [20,24]. Although the photobleaching is a suitable approach to obtain a static snapshot of the cellular composition (in terms of lipids, pigments, etc.), this procedure hinders all metabolic processes and does not allow us to analyze any photochemical reactions occurring in the photosynthetic microorganisms.

Herein, we present a single-cell Raman microscopy assay, which exploits conventional confocal Raman in the visible wavelength-range and does not require any specific equipment or pre-treatment of the bio-specimen. The possibility to track simultaneously Raman signals of both carbon nanotubes [16] and chemical species inside the subcellular compartments [20,25,26,27] can bring noteworthy development in in vivo methodologies to survey the entry of the nanotubes into plant type cells, and study the interaction between the SWCNTs and surrounding cell components. To the best of our knowledge, this is the first repost using Raman spectroscopy to track CNTs inside algae and to survey their interaction with the photosynthetic machinery. This achievement could deepen the knowledge on the relationship between the physicochemical properties of the nanotubes and their biological effects in microalgae, supporting the development of biotechnological applications of SWCNTs. Furthermore, the outcome of this study highlights the possibility to exploit SWCNTs as spectroscopic probes in systems where the fluorescence background of the specimen obstructs the acquisition of Raman scattering signals (Appendix A).

## 2. Materials and Methods

### 2.1. Preparation of SWCNTs

SWCNTs (SG76, CoMoCAT, Chasm Advanced Materials Inc., Canton, MA, USA, carbon content >90%) with an average diameter of 0.9 ± 0.2 nm and an average length of 800 nm (range of 300–2300 nm) were purchased from Sigma Aldrich, and were further purified by mild oxidative thermal treatment at 300 °C for 1 h, to eliminate amorphous carbon nano-powder, which was followed by an acidic treatment with HCl solution (37%) at room temperature for 1 h in order to solubilize the metal catalyst [28]. After the purification step, the average length of the nanotubes was 800 nm, varying between 600 and 1200 nm (the sample population was enriched with long-length nanotubes, Table 1, and Figure S1 in the ESI). After purification, the nanotubes were shortened by tip sonication for 15, 30, or 60 min (3 mm probe, 50% amplitude, VibraCell VC130PB, Sonics&Materials Inc., Newtown, CT, USA) in milli-Q water at 4 °C at a concentration of 20 mg mL^−1^. The shortening procedure improved the homogeneity of sample size distribution with respect to the pristine (as provided by the producer) sample and reduced the average length of the SWCNTs to approximately 600–800 nm (Table 1).

The structural integrity of SWCNTs after the procedures of purification and shortening was evaluated by Raman spectroscopy. While the 15 min shortening preserve the SWCNT structure, the longer treatments of 30 min and 60 min produced nanotubes with compromised sp^2^ network (increase of D band in Raman spectra, Figure S2 in the ESI), which were not included in the further experiments.

Finally, shorten nanotubes were dried in oven at 60 °C for 24 h and the powder was treated as described below. The SWCNTs were dispersed by non-covalent functionalization with single strand DNA (ssDNA), a well-known dispersing agent, which facilitates the stable mono-dispersion of CNTs and enhances their compatibility with biological samples [29,30]. A previously described procedure was adopted[31] using autoclaved (20 min, 121 °C, 1 bar) powder of nanotubes. Briefly, 1 mg of purified SWCNTs (shortened or un-shortened) were dispersed with 2 mg of ssDNA (ss(AT)_15_, Bio-Fab research s.r.l., Rome, Italy) in 1 ml of 0.1 M NaCl by 30 min bath sonication, which was followed by 20 min tip sonication (3 mm tip, at 40% of amplitude) at 4 °C. The mixture was centrifuged at 16,000× *g* for 90 min at 4 °C to remove the big aggregates of nanotubes. The supernatant was further filtered through Amicon^®^ Ultra 100K device (Millipore Sigma, Burlington, MA, USA) to remove free DNA from solution and obtain DNA-wrapped nanotubes dispersed in 10 mM PBS (Phosphate-Buffered Saline, pH 7.4). All procedures were performed under sterile conditions. The concentration of SWCNTs after the filtration was between 40–70 μg mL^−1^ (calculated according to Reference [31]). At each step of the SWCNT manipulation (purification, shortening, and functionalization with DNA), their quality has been controlled by Raman spectroscopy.

### 2.2. Algal Growth and Treatment

The cell-wall deficient mutant (cc400 cw15 mt +) [32,33] of the motile unicellular green alga *Chlamydomonas reinhardtii* was purchased from the Chlamydomonas Resource Center (CRC, University of Minnesota, MN, USA). Axenic cultures of cc400 strain were grown in Tris-Acetate-Phosphate (TAP, pH 7.2) media at 24 °C under continuous illumination of 50 μmol m^−2^ s^−1^ and 150 rpm shaking. All experiments were performed on cultures in the initial-exponential growth phase, characterized with cells density of (3.1 ± 0.17) × 10^6^ cells mL^−1^ and total chlorophyll content of 4.0 ± 0.3 µg mL^−1^. Small aliquots of algae were supplied with DNA-wrapped SWCNTs (unshortened or shortened by 15 min tip sonication) to reach final SWCNT concentration of 1 µg mL^−1^ or 2 µg mL^−1^ and were cultivated under standard conditions for 72 h. Culture growth was monitored by counting cell number per mL by an Automated Cell Counter (TC20, Bio-Rad Laboratories, Inc., Hercules, CA, USA). In each experiment, three separate flasks were set for each concentration treatment. At the end of each trial, 50 μL aliquots from the control and SWCNT-containing cultures were spread on TAP agar medium and colonies were grown for a period of 30 days. The absence of extraneous colonies growing on the agar confirmed the lack of contaminations introduced to the algal cultures via the addition of nanotubes. This point is of critical importance when cell count is used for estimating the growth of algal cultures and CNT phytotoxicity.

### 2.3. Raman Microscopy

For the Raman microscopy assay, (1.5 ± 0.15) × 10^4^ cells (with and without nanotubes) were deposited on Si single crystal wafers. The drops on the Si-wafers containing the algal cells were dried at room temperature until no free water was detected and then the samples were immediately analyzed. Single cells or a group of cells were examined by Horiba eXplora device using a 532 nm laser source and holographic grating of 1200 T with 1 cm^−1^ spectral resolution. In order to avoid cellular damage, the laser power was adjusted by a set of density filters to not exceed 10 mW at the sample surface. 2D positioning of the sample was assured by high-precision-computer-controlled motorized microscope stage with a lateral resolution of 1 × 1 µm when the 100× objective (NA 0.9) was used. The detector was a 1024 × 256 air-cooled CCD. Two-dimensional Raman map of the whole specimen area was obtained by acquiring individual spectra at distinct points of 1 × 1 μm, reducing the overlapping information from adjacent pixels. The acquisition time of each Raman spectrum in the map was 1 s depending on the explored area the acquisition time of a single map was in the range of 100–400 s. First order Raman signal of Si substrate at 520.7 cm^−1^ was used for the calibration of the spectra. The acquisition, processing, analysis, and display of raw data were done via HORIBA Scientific’s LabSpec 6 software (Kyoto, Japan). Principal component analysis (PCA) was performed using a fully integrated Multivariate Analysis (MVA) module powered by Eigenvector Research Inc. (Manson, WA, US) within HORIBA Scientific’s LabSpec 6 software platform. Further analyses and processing of the data including baseline correction, and fitting, etc., were completed using Origin 8.5 (OriginLab, Northampton, MA, USA).

### 2.4. Scanning Electron Microscopy

Scanning electron microscopy (SEM) analyses were performed by using a Hitachi S-4000 Scanning Electron Microscope (Tokyo, Japan). A few microliters of sample dispersion of SWCNTs were deposited on Silicon substrate and an ultra-thin coating of gold was applied to increase the signal-to-noise ratio. SEM images were analyzed by ImageJ [34,35].

### 2.5. Transmission Electron Microscopy

For Transmission electron microscopy (TEM), (1.35 ± 0.15) × 10^7^ cells were collected by weak centrifugation, washed with nanotubes-free TAP medium, and re-suspended in a 2% low melting point agar dissolved in TAP at 37 °C. The cells in agar were high-speed centrifuged (15,500× *g* for 5 min) in conical tubes and then cooled to 20 °C to allow agar curing. The small tip of the agar block containing algae was excised, cut in pieces, and processed as previously reported [36] with some modifications. The blocks were fixed for 1 h at room temperature with a solution of 2% glutaraldehyde in 0.1 M cacodylate buffer (pH 7.4), post-fixed in 1% osmium tetroxide in 0.1 M cacodylate buffer for 30 min at room temperature, and dehydration in acetone series and embedded in epoxy resin (Epon 812, Hexion Inc., Columbus, OH, USA, Sigma Aldrich). Ultrathin (40 nm) sections with no further counterstaining were visualized by Philips CM12 TEM (Philips, Eindhoven, Netherlands) at 100 KV. Defocus was set in order to have the first zero of the microscope transfer function occurring approximately at 10 nm. More than 30 images were analyzed for every sample prepared.

## 3. Results and Discussion

Mono-dispersed or small bundles of SWCNTs easily overcome the cell wall and the membrane of plant type cells (plant, algae, and cyanobacteria) [8,11]. The exact mechanism of SWCNT passage through the cellular barriers is still under debate. However, it seems that the dimensions and surface charge of the nanotubes are among the main determinant factors [8,11]. In the case of the cc400 *Chlamydomonas* mutant, the entry of SWCNTs into the cell could be further facilitated due to the minute amount of the cell wall produced by this strain [32,33]. The low concentrations, 1 or 2 μg mL^−1^ of well-dispersed and purified SWCNTs used in this study did not provoke the formation of visible cell agglomerates and, in the case of shortened nanotubes, did not influence cell proliferation for the 72 h of exposure (Figure S3 in the ESI). A similar lack of toxicity of CNTs when the nanotubes were introduced into the growth medium at a low concentration and high dispersion state has already been observed. The phytotoxic effects of CNTs on algae are mainly ascribed to the ability of nanotubes to initiate cell agglomeration, hinder nutrient uptake, leach metal catalyst residues, or cause oxidative stress damage [11]. The extent to which these effects are pronounced depends on multiple factors such as dimensions, functionalization, dispersion state, and purity level of the nanotubes in addition to their concentration in the medium. We have exploited stably dispersed SWCNTs with a high purity level and small diameter in order to minimize the possible phytotoxicity of the nanomaterials [11]. The unshortened SWCNTs tends to hinder the culture growth and induce a transient slowdown in cell proliferation at 48 h of exposure, which disappeared in the following 24 h (Figure S3 in the ESI). In bacteria, it was demonstrated that long-length SWCNTs (length between 1000 and 5000 nm) were more prone to form aggregates with the cells and had a higher antimicrobial activity that the short-length nanotubes (length <1000 nm) [37]. The population of unshortened SWCNTs was enriched with long-length nanotubes (length > 1000 nm, Figure S1) that most likely promoted the formation of microaggregates with the algal cells hindering their proliferation.

**Figure 1:**
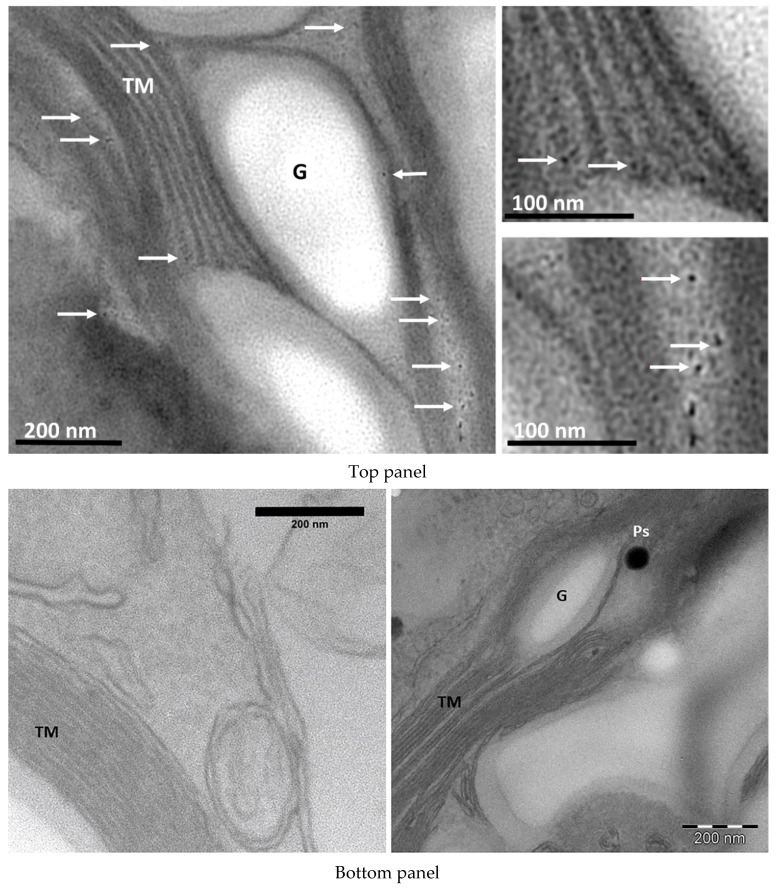
**Top** panel: TEM image of DNA-wrapped SWCNTs (white arrows) located in the stroma and thylakoid membranes of algal chloroplast. **Bottom** panel: chloroplast compartment of cells not exposed to SWCNTs. G–starch grain, TM–thylakoid membranes, Ps-plastoglobules.

The cellular internalization and spatial distribution of the SWCNTs were first proven by TEM using an optimized protocol to promote the visualization of nano-sized carbon-based materials inside the algal cell. TEM images showed a well-conserved morphology of algal chloroplasts with starch granules and thylakoid membranes [38,39] (Figure 1) that were not altered by the treatment with nanotubes. TEM images of *Chlamydomonas* whole cell and details of the chloroplast compartment are shown in Figure S4 in the ESI. The SWCNTs (electron dense formations approximately 10 nm in diameter) were observed in both granal and stromal compartments of the chloroplast of algae grown in the presence of nanotubes, while no similar optically-dense formations were found in the control samples (Figure 1 in the bottom panel and Figure S4 in the ESI).

**Figure 2:**
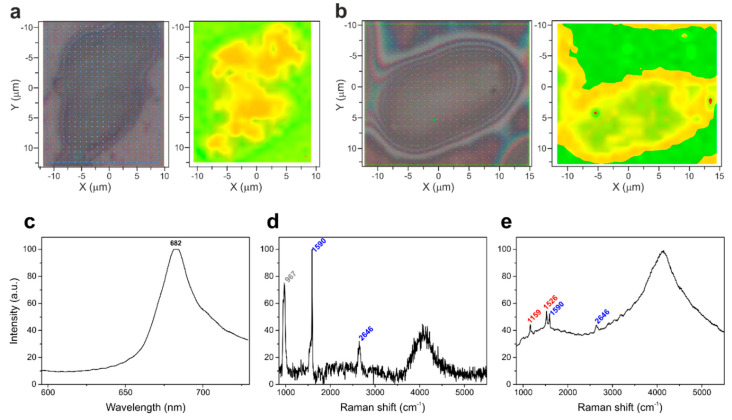
A bright field microscopy images and the corresponding PCA-false-color pictures of Raman maps of control Chlamydomonas cell (**a**) and cell from the culture enriched with 2 µg of SWCNTs mL^−1^ (**b**). The main signals found in Raman maps of cells hosting SWCNTs: (**c**) chlorophyll fluorescence emission, the spectral interval corresponds to 2140-5100 cm^−1^ in the Raman spectra, (**d**) second-order Raman spectrum of silicon (967 cm^−1^) and G band (1590 cm^−1^) and G’ band (2646 cm^−1^) of SWCNTs. (**e**) Raman spectrum with the resonance peaks of carotenoids (numbers in red) and SWCNTs (numbers in blue), and chlorophyll fluorescence background.

2D Raman mapping was performed on cells deposited on Si single-crystal-wafers. These hyperspectral images, in which each x and y spatial coordinate (a pixel) corresponded to a single Raman spectrum, were subjected to PCA to distinguish the spatial distribution of the chemical composition of the specimen [40]. In the case of samples without SWCNTs, the PCA maps displayed two main components (Figure 2a), characterized by the highest degree of variability that can be attributed to the Raman signal of silicon substrate (green region) and the cell fluorescence (yellow region), respectively. Whereas, in the case of algal cells with SWCNTs (Figure 2b), the PCA indicated an additional component whose spectral characteristics belong to the Raman signal of SWCNTs (red spots inside the cell).

Figure 2c–d presents the main signals identified in the Raman maps of cells hosting SWCNTs. Among them, the most intense was the fluorescence emission, centered at approximately 682 nm (Figure 2c), and originating mainly from the chlorophyll pigments of photosynthetic antenna complexes of Photosystem II (PSII) [41]. The SWCNTs were easily distinguishable by their characteristic resonance G band at 1590 cm^−1^ and second order band at 2646 cm^−1^ (Figure 2d) [19]. The Raman spectra of cellular carotenoids (two main peaks centered at 1159 cm^−1^ and 1526 cm^−1^, Figure 2e, and signals of other cellular components (e.g., polyphosphates and starch, Figure S5 in the ESI) were also identified [20,21,22,23].

**Figure 3:**
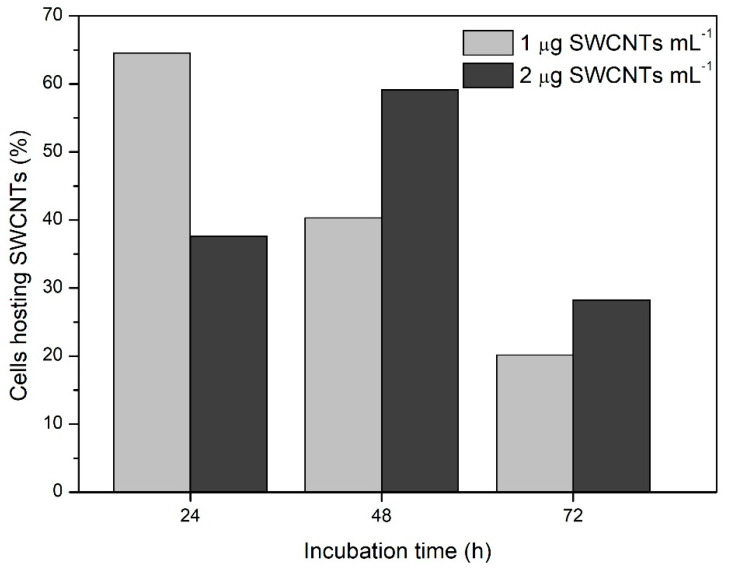
Dynamics of the nanotubes internalization in *Chlamydomonas* cells exposed to low concentrations of shorten SWCNTs. The histogram reports the number of cells, whose Raman map reported at least one point with a typical nanotube-fingerprint (G and G’ band), presented as a percentage of the total number of counted cells. More than 80 cells per single point were scanned. Representative data from a single experiment with three technical repetitions are shown.

The unambiguously distinguishable pattern of the SWCNT-Raman signal endorsed quantitative analyses of the internalization dynamics of SWCNTs into *Chlamydomonas* cells during the 72 h-incubation period. The cellular internalization capacity of the unshortened SWCNTs was very low. In these experiments, most of the nanotubes were localized in the extracellular space and cells hosting one or more SWCNTs were only sporadically found. The shortening procedure enhanced the ability of the SWCMNTs to enter the cells and allowed the collection of statistically meaningful data. In this case, in more than 60% of the mapped cells, the G and G’ Raman bands of at least one nanotube were detected. The maximum number of cells hosting nanotubes was reached after 24 h and 48 h of incubation with 1 µg mL^−1^ and 2 µg mL^−1^ SWCNTs, respectively (Figure 3). The difference between the two treatments was attenuated after 72 h of incubation when the number of cells containing nanotubes was reduced by approximately 50%. So far, it is difficult to argue about the effect of SWCNT concentration on their internalization and/or accumulation in the cells. Nonetheless, it is reasonable to associate the reduction of SWCNTs-containing cells with the cell proliferation over time. Although we acknowledge the need for further and more comprehensive analyses of this phenomenon, the obtained results pointed out an important aspect to be considered in the development of material-based applications in algal biotechnology. Among others, the maintenance of stable effective concentration of the nanomaterials in continuously growing microalgal cultures stands out.

**Figure 4:**
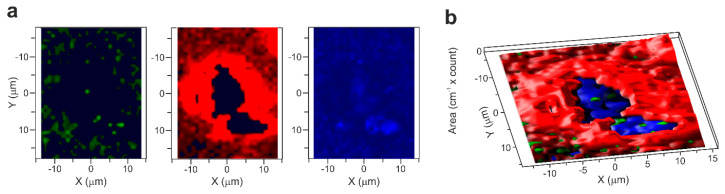
Image reconstruction of a single *Chlamydomonas* cell hosting SWCNTs based Raman spectroscopy signals. (**a**) The left panel (in green) reports the occurrence of G and G’ Raman signal of the SWCNTs inside the algal cell (the reported spectral region, 2600–2700 cm^−1^, is from G’ band), the central panel (in red) presents chlorophyll fluorescence signal (spectral region 3200–4800 cm^−1^), and the right panel (in blue) shows Raman signal originating from carotenoids (spectral region 1000–1530 cm^−1^). (**b**) 3D reconstruction of a cell combining fluorescence emission (red) with Raman signals of SWCNTs (green) and carotenoids (blue).

The confocal Raman microscopy allowed the reconstruction of a map of the spectral signals of an individual algal cell. In cells free of nanotubes, the maps were exclusively populated by a single and very strong signal attributed to the fluorescence of the chlorophyll pigments (Figure S6 in the ESI). The picture was very different when SWCNTs were localized inside the cells. Figure 4a shows the Raman signal of the SWCNTs inside an algal cell (green spots), the chlorophyll fluorescence signal (red region), and the Raman signal originating from carotenoids (blue region). The detection of SWCNT-Raman-fingerprints inside the cell was accompanied by quenching of the fluorescence emission in a proximity to the nanotubes, which allowed the recording of Raman scattering of the underlying chemical constituents of the cell (Figure 4b, Figure S5 in the ESI) [42]. The pseudo-enhancement of the Raman signals observed in these circumstances can be rationalized by hypothesizing electron or excitation energy transfer between the excited chlorophyll molecules and the SWCNTs. This interaction seems to occur in a manner very similar to the mechanism that causes the chemical effects in SERS (Surface-enhanced Raman spectroscopy). The origin of SERS effects in general is explained by at least two mechanisms of electromagnetic and chemical origin [43]. The electromagnetic effect is due to an increase of the electrostatic field in the vicinity of metal nanoparticles or rough surfaces, caused by excitation of surface plasmon-polaritons from the incoming electromagnetic waves. The chemical effects may result from an increase in polarizability of an adsorbate due to charge-transfer (or bond formation) between the fluorescence species and a species acting as an electron acceptor (in our case, carbon nanotubes). The chlorophyll fluorescence quenching mediated by the SWCNTs observed in our study might be explained by the chemical SERS mechanism, i.e., SWCNTs can promote an alternative, non-radiative pathway for deactivation of the chlorophyll singlet excited states, whose functioning allows the detection of Raman scattering of the chemical species close to the nanotubes.

This mechanism of amplification of the Raman signal has a neglected history. It was already highlighted in 1994 by Kagan and McCreery demonstrating that Raman spectra of normally fluorescent materials were obtained by quenching of their fluorescence emission through an adsorption on carbon surfaces [44]. To date, this methodology has been successfully reproduced using graphene-based substrates [45] and our results provided evidence that this mechanism is also valid in bio-hybrid systems containing carbon nanotubes. SWCNTs are known as very effective fluorescence quenchers [46]. Damping of a fluorescence signal induced by SWCNTs has been reported for a variety of fluorophores, such as porphyrins [47], different chemical chromophores [48,49,50], quantum dots [51], and conducting polymers [52]. These mechanistic studies underline mainly an excitation energy transfer or an electron transfer as possible mechanisms that govern the high quenching efficiency of the SWCNTs. It should be pointed out that, in the photoinduced charge transfer between an excited fluorophore and CNTs, the nanotubes may act as both an electron acceptor and donor. The effective behavior depends, among others, on the intrinsic properties of the fluorophore, the conjugation mode of “donor-acceptor” pair, and the local environment [50,53,54].

The scientific literature offers a limited number of studies dealing with CNT interaction with isolated chlorophyll molecules [55], photosynthetic reaction centers (RCs) [56,57], thylakoid membranes [6,58], or isolated light-harvesting complexes [58]. Due to the different experimental conditions exploited in these studies, various possible interaction mechanisms have been identified, including both energy or charge transfer forward and from the nanotubes. In other studies, the reduction of fluorescence emission originating from photosynthetic complexes has been attributed to the occurrence of charge transfer [58] or excitation energy transfer [59] toward the nanotubes. In the latter case [59], the deposition on SWCNT-films of isolated peridinin-chlorophyll-protein complexes, that are energy-harvesting complexes not capable of performing charge-separation, resulted in almost 90% quenching of the fluorescence signal. Taking into account that the Raman measurements in the present study have been done on air-dried cells deposited on Si-wafers, we may assume a significant reduction of the functional PSII RCs able to perform charge separation [60,61,62]. Under these conditions, it is reasonable to hypothesize that the majority of light-harvesting antennae (where most chlorophylls are concentrated) are not functionally coupled to RCs, and promptly transfer their excitation energy from one antenna complex to another and, finally, to the nearby SWCNTs, which serve as energy sinks. This results in vanishing of the chlorophyll fluorescence background in close proximity of the nanotubes. However, the contribution of electron transfer between the PSII RC photoactive pigment (P_680_) and the SWCNTs to the induction of non-radiative relaxation of the excitation could not be completely ruled out. This mechanism assumes accumulation of P_680_^+^, which is a strong fluorescence quencher [63] and generated due to electron leakage toward the SWCNTs, hindered reduction of P_680_^+^ due to the deactivation of water splitting at the PSII donor side during dehydration [62]. Most likely, both mechanisms conferred for the observed fluorescence quenching, even though, under our conditions, the contribution of the energy transfer significantly outdid the electron transfer.

## 4. Conclusions

We provided a novel experimental approach suitable to support nanomaterial-based applications promoting algal and plant biotechnology, and risk assessment studies on CNT phytotoxicity. The strategy we considered in this case employs the robust and cost-affordable conventional Raman to track the cellular internalization of SWCNTs. This approach also bears the potential to advance the understanding of the relationships among physicochemical features of CNTs and their cellular internalization capacity and ability to interact with the adjacent cell components *in vivo*. Furthermore, it represents a handy and label-free tool to track the delivery of genes, therapeutics, pesticides, or fertilizers loaded on carbon nanotubes. We demonstrated SWCNTs supported non-radiative deactivation of excited chlorophyll molecules, resulting in an efficient quenching of their fluorescence emission and enabling the recording of Raman signals of different chemical constituents of the cell using simple spectroscopy and experimental set-up. Thus, SWCNTs may be proposed as effective in vivo spectroscopic probes, able to vanish the fluorescence background of the photosynthetic specimen usually affecting visible spectroscopic analyses. This finding opens new opportunities for exploiting the visible Raman spectroscopy in the biochemical-composition analysis and imaging of living algal cells. This approach may be expanded to all systems where the fluorescence background hinders the acquisition of Raman scattering signals.

Electronic Supplementary Information available (SWCNT length, TEM of control samples, growth curves of *Chlamydomonas* in presence of SWCNTs, Raman signals of cellular components, Raman map of control samples).

## Figures and Tables

**Table 1:** Morphological characterization of SWCNTs after the individual steps of purification, shortening, and functionalization with DNA. The data for pristine tubes refer to the SG76 sample (CoMoCAT, Sigma Aldrich) provided by the producer. Mean values ±SD, derived from size distribution histograms obtained by SEM (n > 200) are presented.

SWCNTs	Length (±SD), nm
**Pristine SWCNTs**	Average 800
(refer to product specifications)	(range 300–2300)
**Purified unshortened SWCNTs**	800 ± 200
**Purified shortened SWCNTs** tip sonication applied for 15 min	710 ± 100
tip sonication applied for 30 min	700 ± 110
tip sonication applied for 60 min	650 ± 150

**Figure A1:**
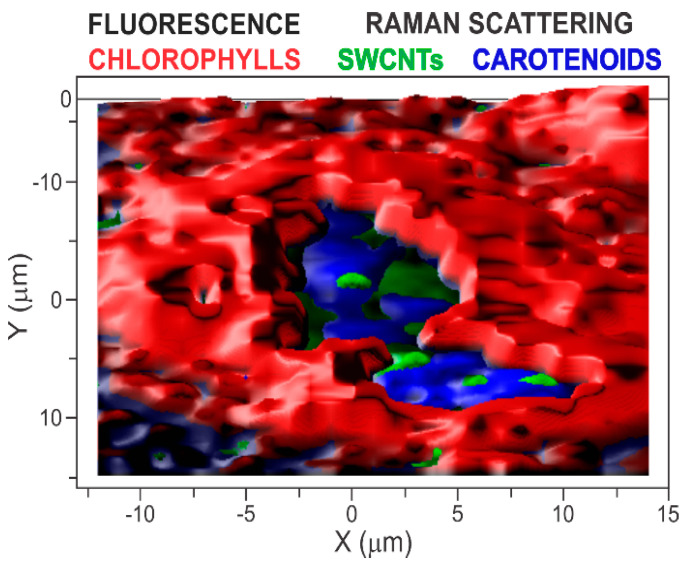
A single-cell Raman microscopy imaging of carbon nanotubes in photosynthetic samples set up to survey nanotubes’ cellular uptake and their interaction with the intracellular environment, demonstrates a non-radiative deactivation of excited chlorophyll molecules supported by the nanotubes. The efficient quenching of the chlorophyll fluorescence permits the detection of Raman signals of several cellular components, including carotenoids, lipids, and carbohydrates.

## Supplementary Materials

The following are available online at https://www.mdpi.com/1996-1944/13/22/5121/s1. Figure S1: SEM characterization of SWCNT length. Figure S2: Raman spectra of purified and shortened SWCNTs. Figure S3: Growth curves of algal cultures exposed to different concentrations of SWCNs. Figure S4: TEM images of control algal cells. Figure S5: Raman spectra detected in algal cells hosting SWCNTs. Figure S6: A bright field microscopy image and the corresponding Raman map of an algal cell free of nanotubes.
