# Supplementary material for: Mapping Single Walled Carbon Nanotubes in Photosynthetic Algae by Single-Cell Confocal Raman Microscopy
Source: Materials (Basel). 2020 Nov 13;13(22):5121. doi: 10.3390/ma13225121 (PMC7698160; doi:10.3390/ma13225121)
Supplement: Supplementary file 1 [file materials-13-05121-s001.pdf]

Mapping Single Walled Carbon Nanotubes in Photosynthetic Algae by Single-Cell Confocal Raman Microscopy

Silvia Orlanducci[1,2,*], Gianluca Fulgenzi[3,4], Andrea Margonelli[2], Giuseppina Rea[2], Taras K. Antal[5,6] and Maya D. Lambreva[2*]

[1] Department of Chemical Science and Technology, University of Rome ''Tor Vergata'', 00133 Rome, Italy
[2] Institute of Crystallography, National Research Council of Italy, 00015 Monterotondo Stazione, RM, Italy, andrea.margonelli@ic.cnr.it (A.M.); giuseppina.rea@ic.cnr.it (G.R.)
[3] Department of Molecular and Clinical Sciences, Faculty of Medicine and Surgery, Marche Polytechnic University, 60126 Ancona, Italy, gianluca.fulgenzi2@nih.gov (G.F)
[4] Mouse Cancer Genetics Program, Center for Cancer Research, National Cancer Institute, Frederick, MD 21702-1201, USA
[5] Department of Biophysics, Faculty of Biology, Lomonosov Moscow State University, 119992 Moscow, Russian Federation taras_an@mail.ru
[6] Laboratory of Integrated Environmental Research, Pskov State University, Pskov, 180000, Russian Federation,
* Corresponding author: silvia.orlanducci@uniroma2.it (S.O.); maya.lambreva@cnr.it (M.D.L.)

Supporting information

1. Morphological and Structural Characterization of Single Walled Carbon Nanotubes (SWCNTs)

Morphological characterization of nanotubes was performed by Scanning Electron Microscopy (SEM). For SEM analyses, the SWCNTs were deposited on Si wafer and metalized. SEM analyses were performed by using Hitachi S-4000 Scanning Electron Microscope. A few microliters of sample dispersion of SWCNTs were deposited on the Silicon substrate and an ultra-thin coating of gold was applied to increase the signal-to-noise ratio. The length of the nanotubes was determined by analyzing SEM images using ImageJ software, as described earlier [1].

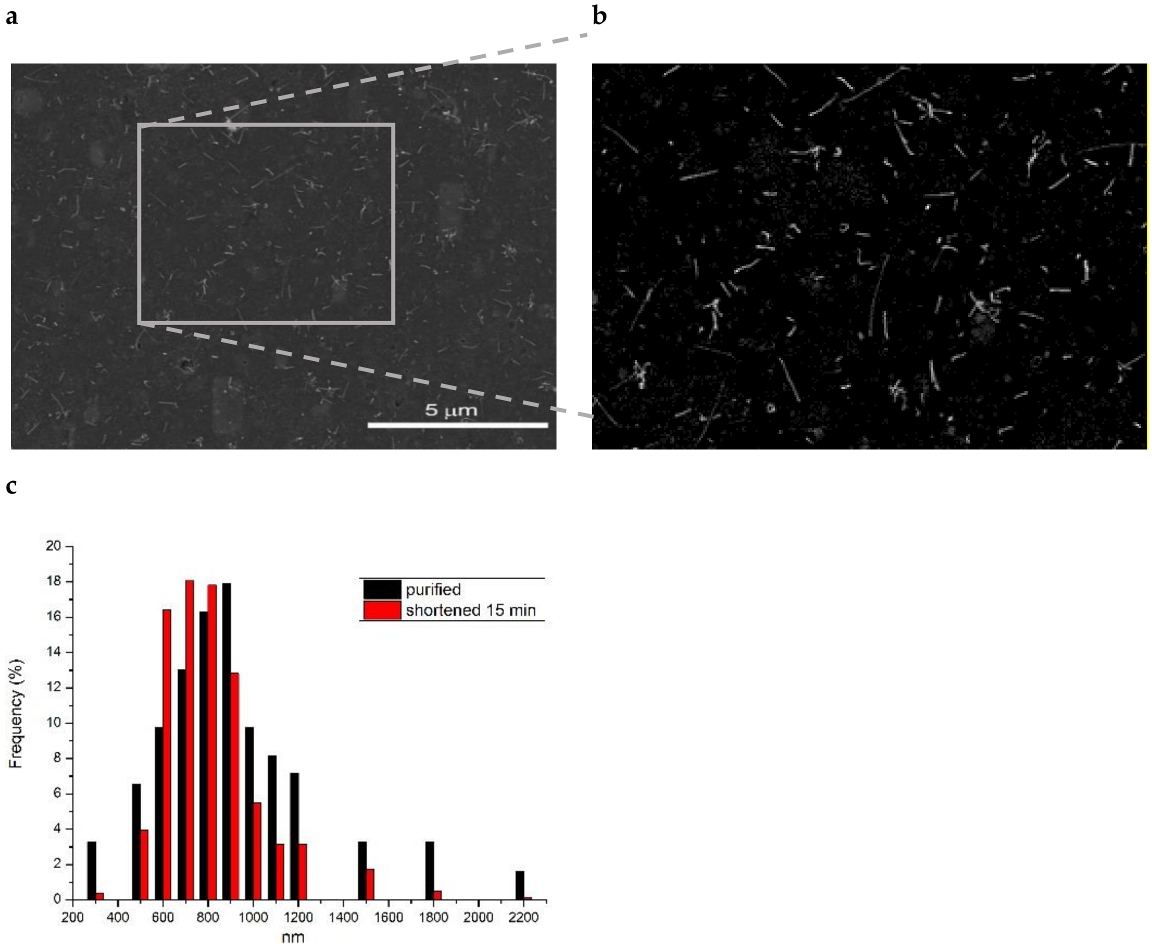

Figure 1. SEM characterization of SWCNT length after the purification and shortening. (**a**) A representative SEM image used in the length estimation of SCNTs. (**b**) A portion of the same SEM image after processing in ImageJ[1]. (**c**) Length distribution histogram of purified unshortened SWCNTs and SWCNTs shortened by 15-min tip sonication.

The structural integrity of SWCNTs after the shortening procedure (15, 30, and 60 min of tip sonication) was estimated by Raman spectroscopy (Figure S2). The 15-min shortening preserve the structure of the nanotubes, while the longer treatments of 30 min and 60 min produced nanotubes with compromised sp^2 network (increase of D band, Figure S2) that were not included in the further experiments.

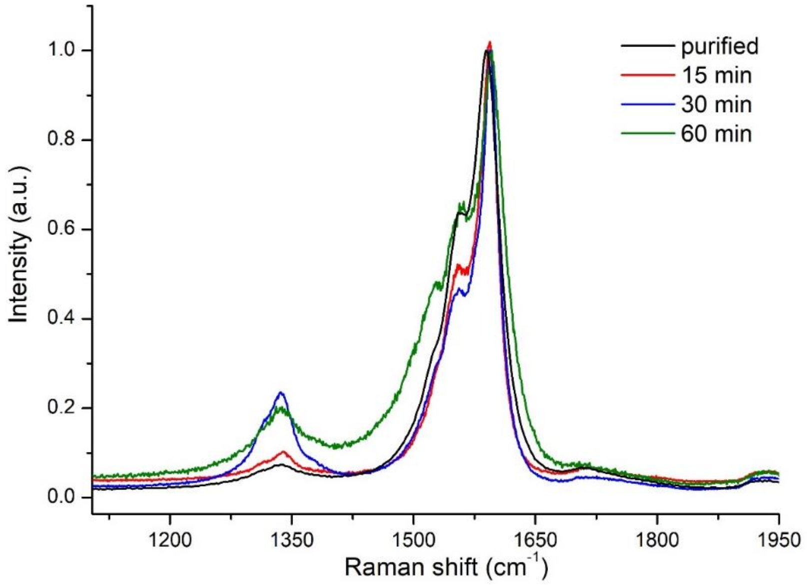

Figure 2. Raman spectra, in the 1200-1900 cm $^{-1}$ spectral range, of purified SWCNTs and after shortening by tip sonication for 15, 30, and 60 min. D band at about 1350 cm^{-1} and G band at about 1580 cm^{-1}. The spectra were collected by using a Horiba eXplora device using a 532-nm laser source.

2. Growth Curves of cc400 *Chlamydomonas* Mutant Exposed to SWCNTs

Algal cultures in initial-exponential growth phase ($3.1 \pm 0.17 \times 10^6$ cells mL^{-1}) were exposed to 1 or 2 μg mL^{-1} of DNA-wrapped SWCNTs, shortened or not. Control and SWCNTs-enriched *Chlamydomonas* cultures were grown for a period of 72 h and culture growth were examined every 24 h. Neither of the treatments induced formation of visible cell agglomerates. However, the treatment with unshortened SWCNTs slowed down cell proliferation during the first 48 h in a concentration-dependent manner.

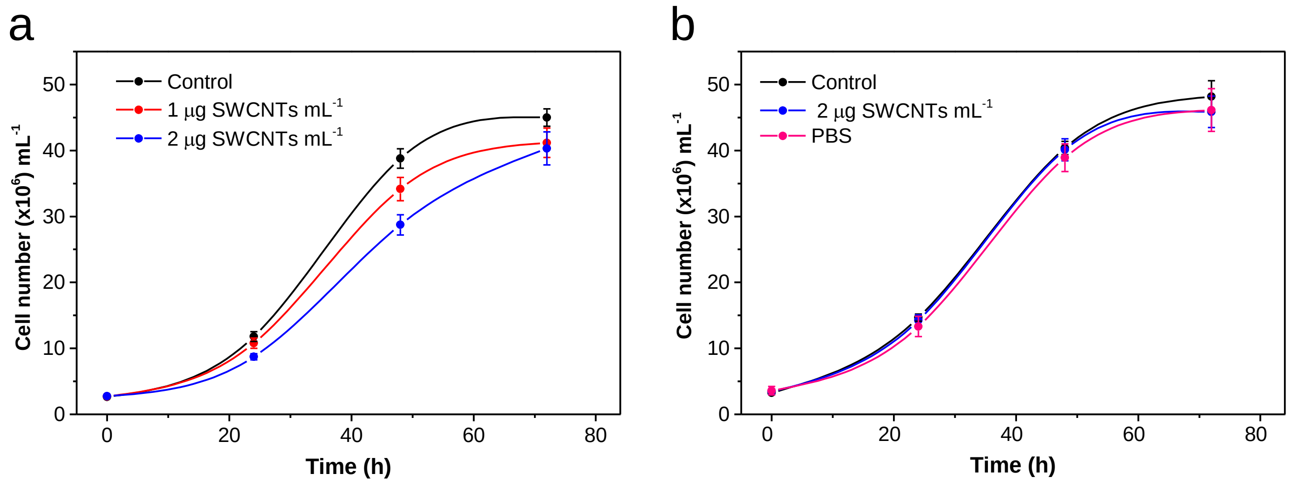

Figure 3. Growth curves of cc400 *Chlamydomonas* cultures exposed to 0, 1, and 2 μg SWCNTs mL^{-1} for a period of 72 h. (**a**) Unshortened SWCNTs. (**b**) SWCNTs shortened by 15 min tip-sonication. The effect of PBS buffer (Phosphate-Buffered Saline) was also tested, as it is present in the SWCNT dispersions. Average values from three separate cultures, with 2-4 technical repetitions for each culture, ±SE.

3. TEM Images of Control Cells

Approximately half of the volume of the oval *C. reinhardtii* cell is occupied by a single cup-shaped chloroplast, which expands towards the cell-wall engirding a "hollow" central part containing the main cellular organelles.[2] The chloroplast rim is in close proximity of the cell wall and plasmalemma membrane. In the case of cc400 mutant, the lack of well-structured cell wall results in cells with an irregular shape when imaged by TEM [3].

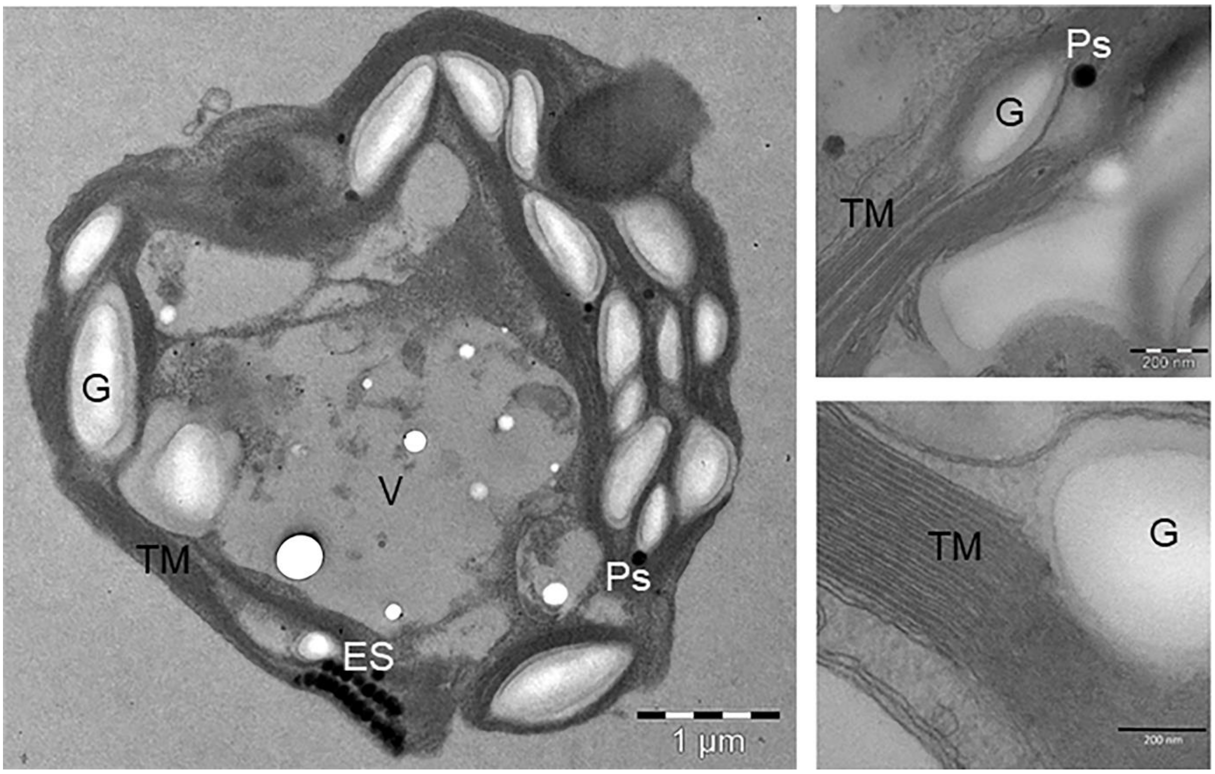

Figure 4. TEM images of a cc400 *Chlamydomonas* mutant and chloroplast of cells not exposed to SWCNTs. The samples have been prepared from control algal cultures from the same growth phase as the one grown in the presence of nanotubes. Well-developed grana stacks and stromal thylakoids (TM) along with plastoglobules (Ps) and abundance of starch granules (G) presented. The whole cell image shows the photoreceptive organelle of Chlamydomonas, namely eyespot (ES).

4. Raman Spectra of Chemical Components Detected in Cells Hosting SWCNTs

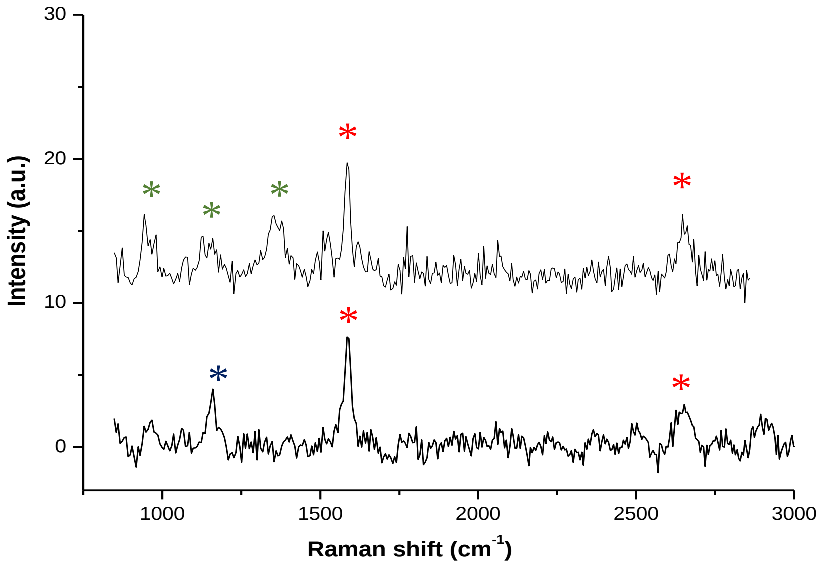

Figure 5. Two Raman spectra detected in *Chlamydomonas* cells hosting SWCNTs: red asterisks denote G and G′ Raman signals of SWCNTs, blue asterisk marks a signal at approximately 1165 cm^{-1} attributed to the polyphosphate species [4] green asterisks mark signals at about 940, 1130, and 1340 cm^{-1} attributed to starch [4].

5. Raman Map of Cells Free of SWCNTs

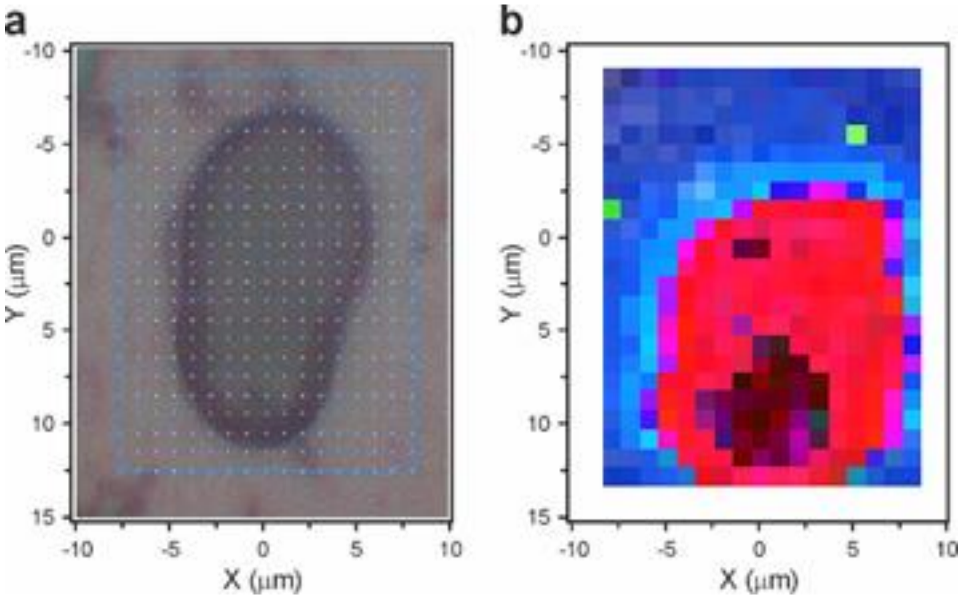

Figure 6. A bright field microscopy image (**a**) and the corresponding Raman map (**b**) of a single *Chlamydomonas* cell free of nanotubes. (**b**) Red and dark red pixels correspond to fluorescence emission of the cell chlorophylls, blue pixels correspond to the Raman signal of Si substrate, and green indicated the Raman signal of a SWCNT localized outside the cell.

References

1. Schneider, C. A.; Rasband, W.S. and Eliceiri K. W. NIH Image to ImageJ: 25 years of image analysis *Nat. Methods*, **2012**, *9*, 671–675.
2. Engel, B.D.; Schaffer,M.; Cuellar,L.K.; Villa, E.; Plitzko, J.M. and Baumeister, W. Native architecture of the Chlamydomonas chloroplast revealed by in situ cryo-electron tomography, *Elife*, **2015**, doi:10.7554/eLife.04889.
3. Davies, D.R. and Plaskitt, A. Cell wall organisation in *Chlamydomonas reinhardi*, *Genet. Res.*, **1971**, *17*, 33–43.
4. Moudříková, Š.; Sadowsky, A.; Metzger, S.; Nedbal, L.; Mettler-Altmann, T.; Mojzeš, P.Quantification of Polyphosphate in Microalgae by Raman Microscopy and by a Reference Enzymatic Assay, *Anal. Chem.* **2017**, *89*, 12006–12013. doi:10.1021/acs.analchem.7b02393.
